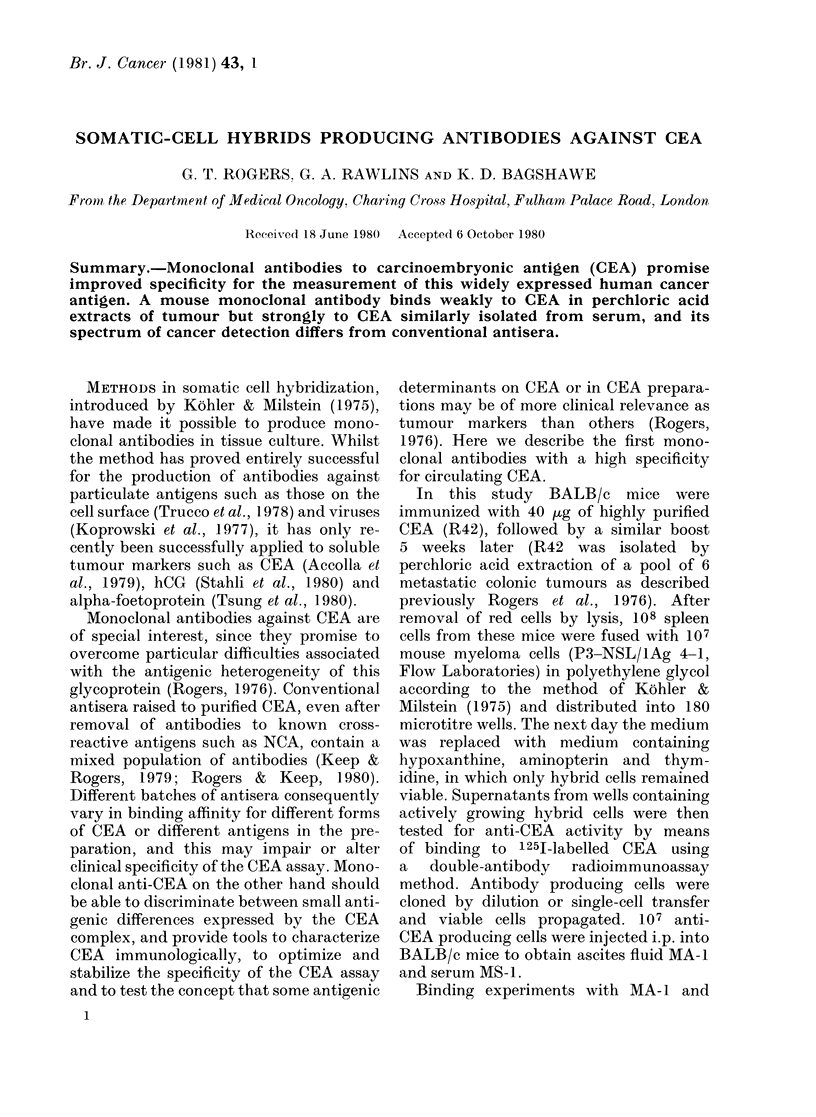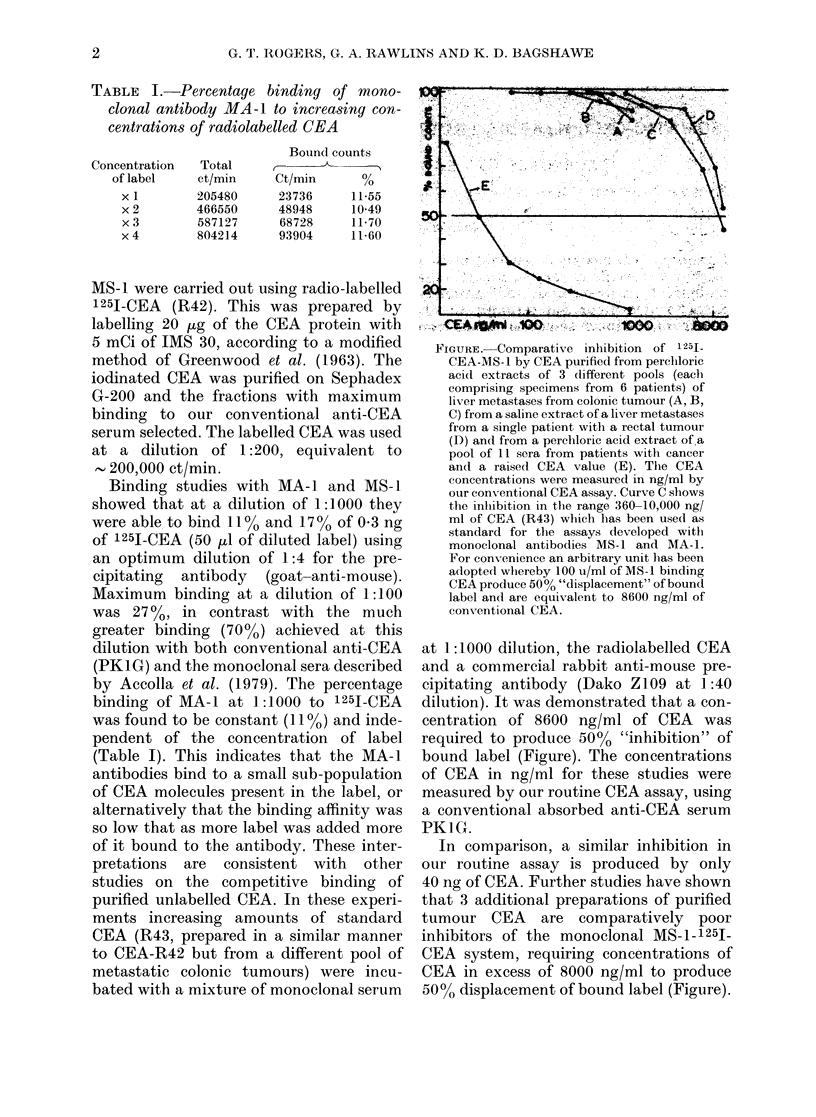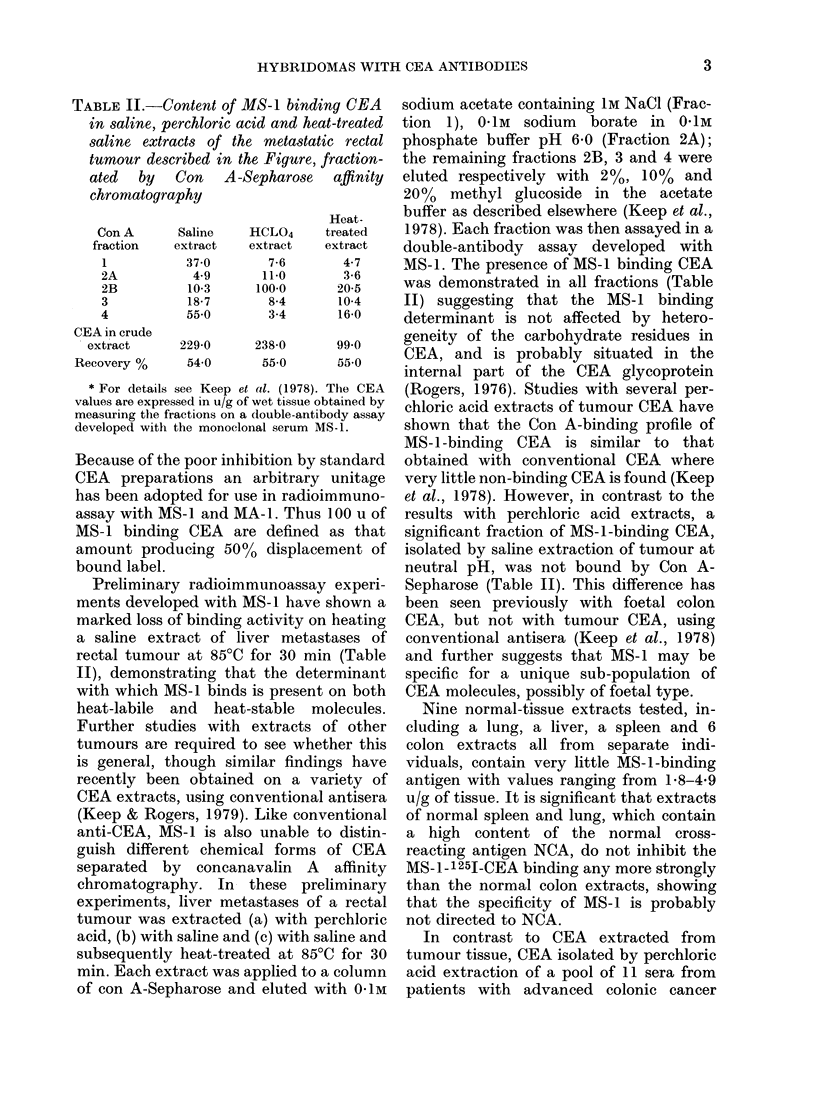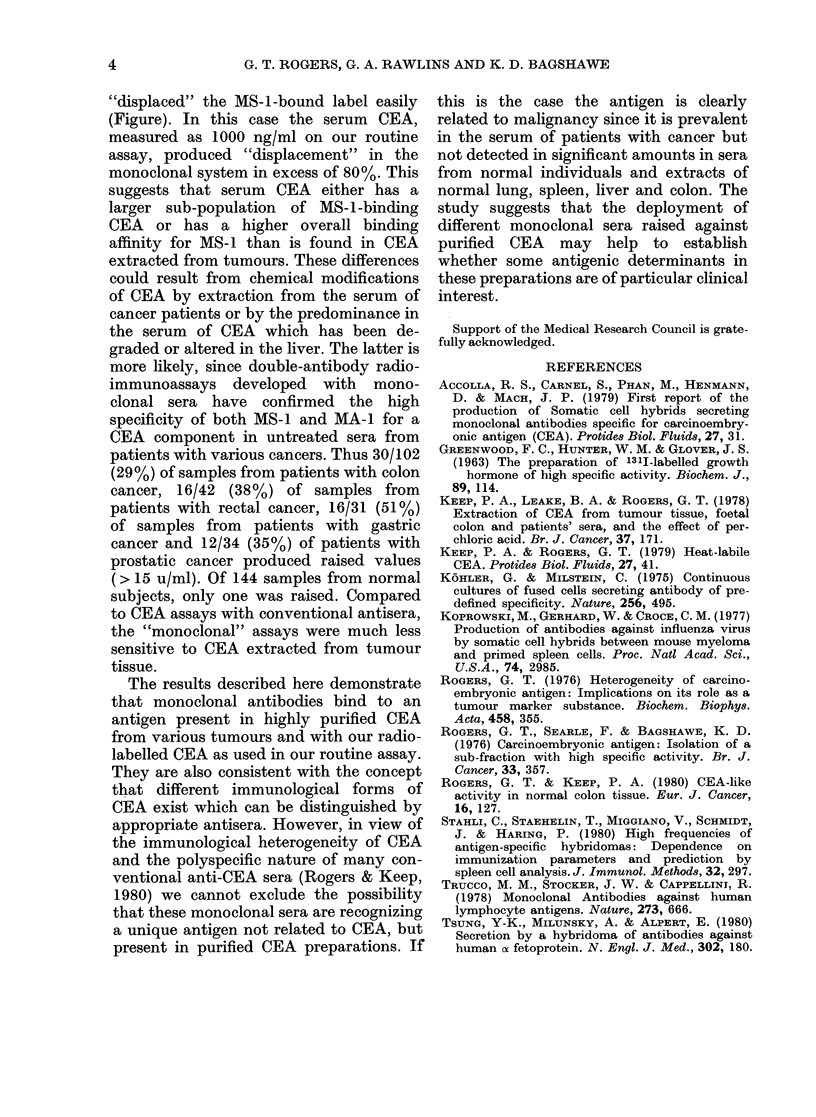# Somatic-cell hybrids producing antibodies against CEA.

**DOI:** 10.1038/bjc.1981.1

**Published:** 1981-01

**Authors:** G. T. Rogers, G. A. Rawlins, K. D. Bagshawe

## Abstract

Monoclonal antibodies to carcinoembryonic antigen (CEA) promise improved specificity for the measurement of this widely expressed human cancer antigen. A mouse monoclonal antibody binds weakly to CEA in perchloric acid extracts of tumour but strongly to CEA similarly isolated from serum, and its spectrum of cancer detection differs from conventional antisera.


*Br. J. Cancer* (1981) **43**, 1

### SOMATIC-CELL HYBRIDS PRODUCING ANTIBODIES AGAINST CEA

G. T. ROGERS, G. A. RAWLINS AND K. D. BAGSHAWE

*From the Department of Medical Oncology, Charing Cross Hospital, Fulham Palace Road, London*

Received 18 June 1980 Accepted 6 October 1980

**Summary.—Monoclonal antibodies to carcinoembryonic antigen (CEA) promise improved specificity for the measurement of this widely expressed human cancer antigen. A mouse monoclonal antibody binds weakly to CEA in perchloric acid extracts of tumour but strongly to CEA similarly isolated from serum, and its spectrum of cancer detection differs from conventional antisera.**

METHODS in somatic cell hybridization, introduced by Köhler & Milstein (1975), have made it possible to produce monoclonal antibodies in tissue culture. Whilst the method has proved entirely successful for the production of antibodies against particulate antigens such as those on the cell surface (Trucco *et al.*, 1978) and viruses (Koprowski *et al.*, 1977), it has only recently been successfully applied to soluble tumour markers such as CEA (Accolla *et al.*, 1979), hCG (Stahli *et al.*, 1980) and alpha-foetoprotein (Tsung *et al.*, 1980).

Monoclonal antibodies against CEA are of special interest, since they promise to overcome particular difficulties associated with the antigenic heterogeneity of this glycoprotein (Rogers, 1976). Conventional antisera raised to purified CEA, even after removal of antibodies to known cross-reactive antigens such as NCA, contain a mixed population of antibodies (Keep & Rogers, 1979; Rogers & Keep, 1980). Different batches of antisera consequently vary in binding affinity for different forms of CEA or different antigens in the preparation, and this may impair or alter clinical specificity of the CEA assay. Monoclonal anti-CEA on the other hand should be able to discriminate between small antigenic differences expressed by the CEA complex, and provide tools to characterize CEA immunologically, to optimize and stabilize the specificity of the CEA assay and to test the concept that some antigenic determinants on CEA or in CEA preparations may be of more clinical relevance as tumour markers than others (Rogers, 1976). Here we describe the first monoclonal antibodies with a high specificity for circulating CEA.

In this study BALB/c mice were immunized with 40 $\mu$g of highly purified CEA (R42), followed by a similar boost 5 weeks later (R42 was isolated by perchloric acid extraction of a pool of 6 metastatic colonic tumours as described previously Rogers *et al.*, 1976). After removal of red cells by lysis, $10^8$ spleen cells from these mice were fused with $10^7$ mouse myeloma cells (P3–NSL/1Ag 4–1, Flow Laboratories) in polyethylene glycol according to the method of Köhler & Milstein (1975) and distributed into 180 microtitre wells. The next day the medium was replaced with medium containing hypoxanthine, aminopterin and thymidine, in which only hybrid cells remained viable. Supernatants from wells containing actively growing hybrid cells were then tested for anti-CEA activity by means of binding to $^{125}$I-labelled CEA using a double-antibody radioimmunoassay method. Antibody producing cells were cloned by dilution or single-cell transfer and viable cells propagated. $10^7$ anti-CEA producing cells were injected i.p. into BALB/c mice to obtain ascites fluid MA-1 and serum MS-1.

Binding experiments with MA-1 and

TABLE I.—*Percentage binding of monoclonal antibody MA-1 to increasing concentrations of radiolabelled CEA*

| Concentration of label | Total ct/min | Bound counts Ct/min | % |
|---|---|---|---|
| ×1 | 205480 | 23736 | 11·55 |
| ×2 | 466550 | 48948 | 10·49 |
| ×3 | 587127 | 68728 | 11·70 |
| ×4 | 804214 | 93904 | 11·60 |

MS-1 were carried out using radio-labelled $^{125}$I-CEA (R42). This was prepared by labelling 20 μg of the CEA protein with 5 mCi of IMS 30, according to a modified method of Greenwood *et al.* (1963). The iodinated CEA was purified on Sephadex G-200 and the fractions with maximum binding to our conventional anti-CEA serum selected. The labelled CEA was used at a dilution of 1:200, equivalent to ~200,000 ct/min.

Binding studies with MA-1 and MS-1 showed that at a dilution of 1:1000 they were able to bind 11% and 17% of 0·3 ng of $^{125}$I-CEA (50 μl of diluted label) using an optimum dilution of 1:4 for the precipitating antibody (goat–anti-mouse). Maximum binding at a dilution of 1:100 was 27%, in contrast with the much greater binding (70%) achieved at this dilution with both conventional anti-CEA (PK1G) and the monoclonal sera described by Accolla *et al.* (1979). The percentage binding of MA-1 at 1:1000 to $^{125}$I-CEA was found to be constant (11%) and independent of the concentration of label (Table I). This indicates that the MA-1 antibodies bind to a small sub-population of CEA molecules present in the label, or alternatively that the binding affinity was so low that as more label was added more of it bound to the antibody. These interpretations are consistent with other studies on the competitive binding of purified unlabelled CEA. In these experiments increasing amounts of standard CEA (R43, prepared in a similar manner to CEA-R42 but from a different pool of metastatic colonic tumours) were incubated with a mixture of monoclonal serum

FIGURE.—Comparative inhibition of $^{125}$I-CEA-MS-1 by CEA purified from perchloric acid extracts of 3 different pools (each comprising specimens from 6 patients) of liver metastases from colonic tumour (A, B, C) from a saline extract of a liver metastases from a single patient with a rectal tumour (D) and from a perchloric acid extract of a pool of 11 sera from patients with cancer and a raised CEA value (E). The CEA concentrations were measured in ng/ml by our conventional CEA assay. Curve C shows the inhibition in the range 360–10,000 ng/ml of CEA (R43) which has been used as standard for the assays developed with monoclonal antibodies MS-1 and MA-1. For convenience an arbitrary unit has been adopted whereby 100 u/ml of MS-1 binding CEA produce 50% "displacement" of bound label and are equivalent to 8600 ng/ml of conventional CEA.

at 1:1000 dilution, the radiolabelled CEA and a commercial rabbit anti-mouse precipitating antibody (Dako Z109 at 1:40 dilution). It was demonstrated that a concentration of 8600 ng/ml of CEA was required to produce 50% "inhibition" of bound label (Figure). The concentrations of CEA in ng/ml for these studies were measured by our routine CEA assay, using a conventional absorbed anti-CEA serum PK1G.

In comparison, a similar inhibition in our routine assay is produced by only 40 ng of CEA. Further studies have shown that 3 additional preparations of purified tumour CEA are comparatively poor inhibitors of the monoclonal MS-1-$^{125}$I-CEA system, requiring concentrations of CEA in excess of 8000 ng/ml to produce 50% displacement of bound label (Figure).

TABLE II.—*Content of MS-1 binding CEA in saline, perchloric acid and heat-treated saline extracts of the metastatic rectal tumour described in the Figure, fractionated by Con A-Sepharose affinity chromatography*

| Con A fraction | Saline extract | $HClO_4$ extract | Heat-treated extract |
|---|---|---|---|
| 1 | 37·0 | 7·6 | 4·7 |
| 2A | 4·9 | 11·0 | 3·6 |
| 2B | 10·3 | 100·0 | 20·5 |
| 3 | 18·7 | 8·4 | 10·4 |
| 4 | 55·0 | 3·4 | 16·0 |
| CEA in crude extract | 229·0 | 238·0 | 99·0 |
| Recovery % | 54·0 | 55·0 | 55·0 |

* For details see Keep *et al.* (1978). The CEA values are expressed in u/g of wet tissue obtained by measuring the fractions on a double-antibody assay developed with the monoclonal serum MS-1.

Because of the poor inhibition by standard CEA preparations an arbitrary unitage has been adopted for use in radioimmunoassay with MS-1 and MA-1. Thus 100 u of MS-1 binding CEA are defined as that amount producing 50% displacement of bound label.

Preliminary radioimmunoassay experiments developed with MS-1 have shown a marked loss of binding activity on heating a saline extract of liver metastases of rectal tumour at 85°C for 30 min (Table II), demonstrating that the determinant with which MS-1 binds is present on both heat-labile and heat-stable molecules. Further studies with extracts of other tumours are required to see whether this is general, though similar findings have recently been obtained on a variety of CEA extracts, using conventional antisera (Keep & Rogers, 1979). Like conventional anti-CEA, MS-1 is also unable to distinguish different chemical forms of CEA separated by concanavalin A affinity chromatography. In these preliminary experiments, liver metastases of a rectal tumour was extracted (a) with perchloric acid, (b) with saline and (c) with saline and subsequently heat-treated at 85°C for 30 min. Each extract was applied to a column of con A-Sepharose and eluted with 0·1M sodium acetate containing 1M NaCl (Fraction 1), 0·1M sodium borate in 0·1M phosphate buffer pH 6·0 (Fraction 2A); the remaining fractions 2B, 3 and 4 were eluted respectively with 2%, 10% and 20% methyl glucoside in the acetate buffer as described elsewhere (Keep *et al.*, 1978). Each fraction was then assayed in a double-antibody assay developed with MS-1. The presence of MS-1 binding CEA was demonstrated in all fractions (Table II) suggesting that the MS-1 binding determinant is not affected by heterogeneity of the carbohydrate residues in CEA, and is probably situated in the internal part of the CEA glycoprotein (Rogers, 1976). Studies with several perchloric acid extracts of tumour CEA have shown that the Con A-binding profile of MS-1-binding CEA is similar to that obtained with conventional CEA where very little non-binding CEA is found (Keep *et al.*, 1978). However, in contrast to the results with perchloric acid extracts, a significant fraction of MS-1-binding CEA, isolated by saline extraction of tumour at neutral pH, was not bound by Con A-Sepharose (Table II). This difference has been seen previously with foetal colon CEA, but not with tumour CEA, using conventional antisera (Keep *et al.*, 1978) and further suggests that MS-1 may be specific for a unique sub-population of CEA molecules, possibly of foetal type.

Nine normal-tissue extracts tested, including a lung, a liver, a spleen and 6 colon extracts all from separate individuals, contain very little MS-1-binding antigen with values ranging from 1·8–4·9 u/g of tissue. It is significant that extracts of normal spleen and lung, which contain a high content of the normal cross-reacting antigen NCA, do not inhibit the MS-1-$^{125}$I-CEA binding any more strongly than the normal colon extracts, showing that the specificity of MS-1 is probably not directed to NCA.

In contrast to CEA extracted from tumour tissue, CEA isolated by perchloric acid extraction of a pool of 11 sera from patients with advanced colonic cancer

"displaced" the MS-1-bound label easily (Figure). In this case the serum CEA, measured as 1000 ng/ml on our routine assay, produced "displacement" in the monoclonal system in excess of 80%. This suggests that serum CEA either has a larger sub-population of MS-1-binding CEA or has a higher overall binding affinity for MS-1 than is found in CEA extracted from tumours. These differences could result from chemical modifications of CEA by extraction from the serum of cancer patients or by the predominance in the serum of CEA which has been degraded or altered in the liver. The latter is more likely, since double-antibody radioimmunoassays developed with monoclonal sera have confirmed the high specificity of both MS-1 and MA-1 for a CEA component in untreated sera from patients with various cancers. Thus 30/102 (29%) of samples from patients with colon cancer, 16/42 (38%) of samples from patients with rectal cancer, 16/31 (51%) of samples from patients with gastric cancer and 12/34 (35%) of patients with prostatic cancer produced raised values (>15 u/ml). Of 144 samples from normal subjects, only one was raised. Compared to CEA assays with conventional antisera, the "monoclonal" assays were much less sensitive to CEA extracted from tumour tissue.

The results described here demonstrate that monoclonal antibodies bind to an antigen present in highly purified CEA from various tumours and with our radiolabelled CEA as used in our routine assay. They are also consistent with the concept that different immunological forms of CEA exist which can be distinguished by appropriate antisera. However, in view of the immunological heterogeneity of CEA and the polyspecific nature of many conventional anti-CEA sera (Rogers & Keep, 1980) we cannot exclude the possibility that these monoclonal sera are recognizing a unique antigen not related to CEA, but present in purified CEA preparations. If this is the case the antigen is clearly related to malignancy since it is prevalent in the serum of patients with cancer but not detected in significant amounts in sera from normal individuals and extracts of normal lung, spleen, liver and colon. The study suggests that the deployment of different monoclonal sera raised against purified CEA may help to establish whether some antigenic determinants in these preparations are of particular clinical interest.

Support of the Medical Research Council is gratefully acknowledged.

### REFERENCES

ACCOLLA, R. S., CARNEL, S., PHAN, M., HENMANN, D. & MACH, J. P. (1979) First report of the production of Somatic cell hybrids secreting monoclonal antibodies specific for carcinoembryonic antigen (CEA). *Protides Biol. Fluids*, **27**, 31.

GREENWOOD, F. C., HUNTER, W. M. & GLOVER, J. S. (1963) The preparation of $^{131}$I-labelled growth hormone of high specific activity. *Biochem. J.*, **89**, 114.

KEEP, P. A., LEAKE, B. A. & ROGERS, G. T. (1978) Extraction of CEA from tumour tissue, foetal colon and patients' sera, and the effect of perchloric acid. *Br. J. Cancer*, **37**, 171.

KEEP, P. A. & ROGERS, G. T. (1979) Heat-labile CEA. *Protides Biol. Fluids*, **27**, 41.

KÖHLER, G. & MILSTEIN, C. (1975) Continuous cultures of fused cells secreting antibody of predefined specificity. *Nature*, **256**, 495.

KOPROWSKI, M., GERHARD, W. & CROCE, C. M. (1977) Production of antibodies against influenza virus by somatic cell hybrids between mouse myeloma and primed spleen cells. *Proc. Natl Acad. Sci., U.S.A.*, **74**, 2985.

ROGERS, G. T. (1976) Heterogeneity of carcinoembryonic antigen: Implications on its role as a tumour marker substance. *Biochem. Biophys. Acta*, **458**, 355.

ROGERS, G. T., SEARLE, F. & BAGSHAWE, K. D. (1976) Carcinoembryonic antigen: Isolation of a sub-fraction with high specific activity. *Br. J. Cancer*, **33**, 357.

ROGERS, G. T. & KEEP, P. A. (1980) CEA-like activity in normal colon tissue. *Eur. J. Cancer*, **16**, 127.

STAHLI, C., STAEHELIN, T., MIGGIANO, V., SCHMIDT, J. & HARING, P. (1980) High frequencies of antigen-specific hybridomas: Dependence on immunization parameters and prediction by spleen cell analysis. *J. Immunol. Methods*, **32**, 297.

TRUCCO, M. M., STOCKER, J. W. & CAPPELLINI, R. (1978) Monoclonal Antibodies against human lymphocyte antigens. *Nature*, **273**, 666.

TSUNG, Y-K., MILUNSKY, A. & ALPERT, E. (1980) Secretion by a hybridoma of antibodies against human α fetoprotein. *N. Engl. J. Med.*, **302**, 180.